# Chronic Adaptation of Achilles Tendon Tissues upon Injury to Rotator Cuff Tendon in Hyperlipidemic Swine

**DOI:** 10.26502/josm.511500146

**Published:** 2024-05-10

**Authors:** Merlin Rajesh Lal, Devendra K Agrawal

**Affiliations:** Department of Translational Research, College of Osteopathic Medicine of the Pacific, Western University of Health Sciences, Pomona, California USA

**Keywords:** Achilles tendon, Hyperlipidemia, Infraspinatus tendon, Rotator cuff injury, Tendon injury

## Abstract

The biomechanical properties of the tendon are affected due to the changes in composition of the tendon extracellular matrix (ECM). Age, overuse, trauma and metabolic disorders are a few associated conditions that contribute to tendon abnormalities. Hyperlipidemia is one of the leading factors that contribute to the compromised biomechanical. Injury was made on infraspinatus tendon of hyperlipidemic swines. After 8 weeks (i) infraspinatus tendon from the injury site, (ii) infraspinatus tendon from the contralateral side and (iii) Achilles tendon, were collected and analyzed for ECM components that form the major part in biomechanical properties. Immunostaining of infraspinatus tendon on the injury site had higher staining collagen type-1 (COL1A1), biglycan, prolyl 4-hydroxylase and mohawk but lower staining for decorin than the control group. The Achilles tendon of the swines that had injury on infraspinatus tendon showed a chronic adaptation towards load which was evident from a more organized ECM with increased decorin, mohawk and decreased biglycan, scleraxis. The mechanism behind the collagen turnover and chronic adaptation to load need to be studied in detail with the biomechanical properties.

## 1. Introduction

Tendon is a dense connective tissue that connects the muscle to bone. The extracellular matrix (ECM) of the tendon tissue is mainly composed of collagen, proteoglycans, glycoproteins, water and cells [1]. The composition and arrangement of the ECM is important in providing stability of the tissue and transferring of load generated by muscles to the bone. This is enabled by the hierarchical arrangement of collagen fibrils embedded with other components of the ECM [2]. Tendon pathology may be caused by traumatic injuries, degenerative diseases or tendinopathy related to overuse. Ligament and tendon injuries account for 50% of musculoskeletal injuries and among those rotator cuff tendon tear require corrective surgeries. Injuries to tendon do not heal by itself [2,3]. More than 50% of sports injuries are related to Achilles tendon and rotator cuff injuries in more than 30% of population above age 60. Various factors such as age, overuse, trauma, and metabolic disorders contribute to the tendon abnormalities, and accompanied by the complex biological regulation of the tendon ECM, an effective treatment is yet to be developed. And hence the regulation and maintenance of the tendon ECM need to be studied in detail [4].

Hyperlipidemia, a systemic disorder, is reported to deposit lipids intercalating the tendons. With time those tendons are compromised structurally and in turn affect the biomechanical properties of the tendons leading to degenerative tare and retear [5–9]. But the underlying mechanism that triggered the change in the composition of the tendon ECM is vastly not evident.

Previously we have reported that hyperlipidemia lowers the biomechanical properties of the rotator cuff tendons using swine models [5,10]. Our aim of this study is to understand the influence of hyperlipidemia in the maintenance and turnover of the tendon ECM that contributes to the biomechanical properties of the tendon. We created injury in the infraspinatus tendon of the rotator cuff in hyperlipidemic swine models and analyzed the expression of (1) collagen type-1, collagen type-3, decorin and biglycan of the ECM, (2) mohawk (MKX) and Scleraxis (SCX), transcription factors and (3) lysyl oxidase (LOX), prolyl 4-hydeozylase (P4HA1) and protein disulphide-isomerase (PDI) in the Infraspinatus tendon and Achilles tendon.

## 2. Materials and Methods

### 2.1 Animals and tendon tissue collection and preparation

The Institutional Animal Care and Use Committee (IACUC) of Western University of Health Sciences, Pomona, CA, USA approved the experimental research protocol (R22IACUC034).

Yucatan miniswine (Sus scrofa) were purchased from Premier Bioresources, Ramona, CA, USA. The swines were acclimatized 12/12 hours of light-dark cycle with feeding twice a day. The swines were separated into three groups. Group-1, Hyperlipidemia (HYP-L) and Group-2, rotator cuff injury (RCI) had four swines each which received cholesterol-high-fat diet, while group-3, control (CONT) had three swines which were fed with normal pig diet. In group-2 swines, rotator cuff injury or injury followed by repair with by Mason-Allen suture technique were made as reported earlier [10–12].

After 8 weeks the swines were sacrificed. Achilles tendon, the infraspinatus tendon from the surgical injury side (IS) and the contralateral side (CS) were collected and kept immersed in 10% buffered formalin until use.

### 2.2 Immunohistochemistry

Immunohistochemistry (IHC) was performed to identify the expression of ECM components. Paraffin embedded tendon tissues from four hyperlipidemic swines and three control swines were sectioned (7μm), deparaffinized, rehydrated and antigen retrieval were done using 0.25% trypsin (wt/v) at room temperature for 15 minutes. The tissue sections were incubated with mouse anti-decorin (NBP3–20641) at 1:50 dilution, mouse anti-biglycan (16409–1-AP) at 1:100 dilution, mouse anti- COL1A1 (ab6308) at 1:50 dilution, rabbit anti- COL3A1 (ab7778), rabbit anti-LOX (PA1–16953) at 1:200 dilution, rabbit anti-P4HA1 (12658–1-AP) at 1:100 dilution, rabbit anti-MKX (PA5–98612) at 1:100 dilution, goat anti-scleraxis (SCX) (sc-87425) at 1:200 dilution, and mouse anti-PDI (MA3–019) at 1:200 dilution, at 4°C overnight. After washing with phosphate-buffered saline (PBS), tissues were incubated with secondary antibody peroxidase-conjugated anti-mouse or anti-rabbit or anti-goat (vector Laboratories, United States) at 1:200 dilution for 1 hour at room temperature. The tissue sections were washed with PBS and color-developed using AEC (3-amino-9-ethylcarbazole), washed with water and counterstained with hematoxylin. Images of the tissue sections were acquired using Leica DM6 microscope with 20X objective. The high-resolution images were analyzed for the mean intensity of staining and %area of staining using Fiji Image J analysis. Five images per sample were used for the analysis.

### 2.3 Statistical analysis

The statistical analyses for the image analysis were performed using one-way analysis of variance (ANOVA) using GraphPad Prism.9.5.1 software. The p-value of < 0.05 was considered statistically significant.

## 3. Results

### 3.1 Immunostaining of infraspinatus tendon

**Figure 1:**
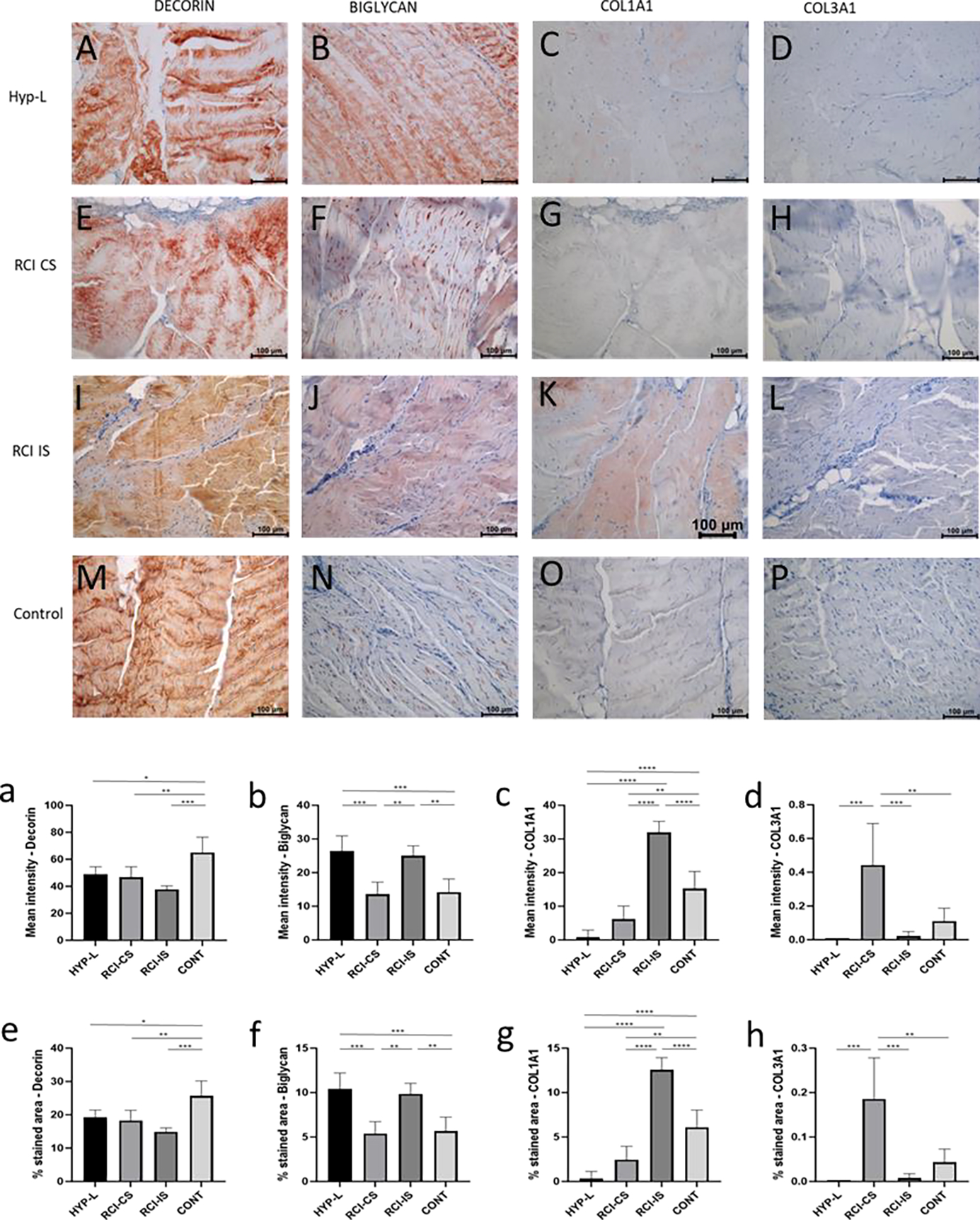
Representative image of immunostaining of infraspinatus tendon for decorin (A,E,I,M), biglycan (B,F,J,N), COL1A1 (C,G,K,O), and COL3A1 (D,H,L,P). Mean intensity of staining and % area of staining of decorin (a,e), biglycan (b,f), COL1A1 (c,g), and COL3A1 (d,h). HYP-L indicates group of swines that received cholesterol-high-fat diet but no surgery. RCI-CS indicates uninjured tendon from contralateral side of swine that underwent either injury or injury + repair surgery and cholesterol-high-fat diet. RCI-IS indicates tendon tissues that had either of the surgery. CONT indicates control group of swines that received normal diet and no surgery. The images were acquired using 20x objective. Values are shown as mean ± SD; n=3–4). * *P*<0.05, ** *P*<0.01, *** *P*<0.001, **** *P*<0.0001.

Immunostaining of infraspinatus tendon of the rotator cuff for decorin, biglycan, collagen-type 1 (COL1A1), and collagen-type 3 (COL3A1) are shown in Figure 1. Tendon tissue from all the three groups showed positive staining for the decorin and biglycan. Semiquantitative analysis of the intensity of staining and % area stained revealed that the control group had more decorin (Figure 1a and 1e) than the hyperlipidemia swines (HYP-L), contralateral tendons of the surgery group (RCI-CS), injured group (RCI-IS). The mean intensity of staining in the HYP-L group and the RCI-CS had no difference but were significantly lower than the control group of swines. On the other hand, positive staining for biglycan was seen in all group of swines. But the HYP-L swines and the contralateral tendon of the injury group showed higher positive staining for biglycan than the injured tendons and control tendons.

Collagen-1 (COL1A1) positive staining varied among all the groups. RCI-IS group of tissue had the highest positivity to COL1A1 while HYP-L group of tissues had the lowest positive staining for the same. Infraspinatus tendon tissues from RCI-CS and HYP-L group of tissues had very low positive staining for COL1A1 as evident form the mean intensity and % area of staining quantification (Figure 1c and 1g). The difference in mean intensity of staining and staining area between the control group of tissues, RCI-CS and HYP-L group were significant. Meanwhile Collagen-3 (COL3A1) positive staining was observed prominent in the RCI-CS group (Figure 1d and 1h) which is higher than control group of swine.

**Figure 2:**
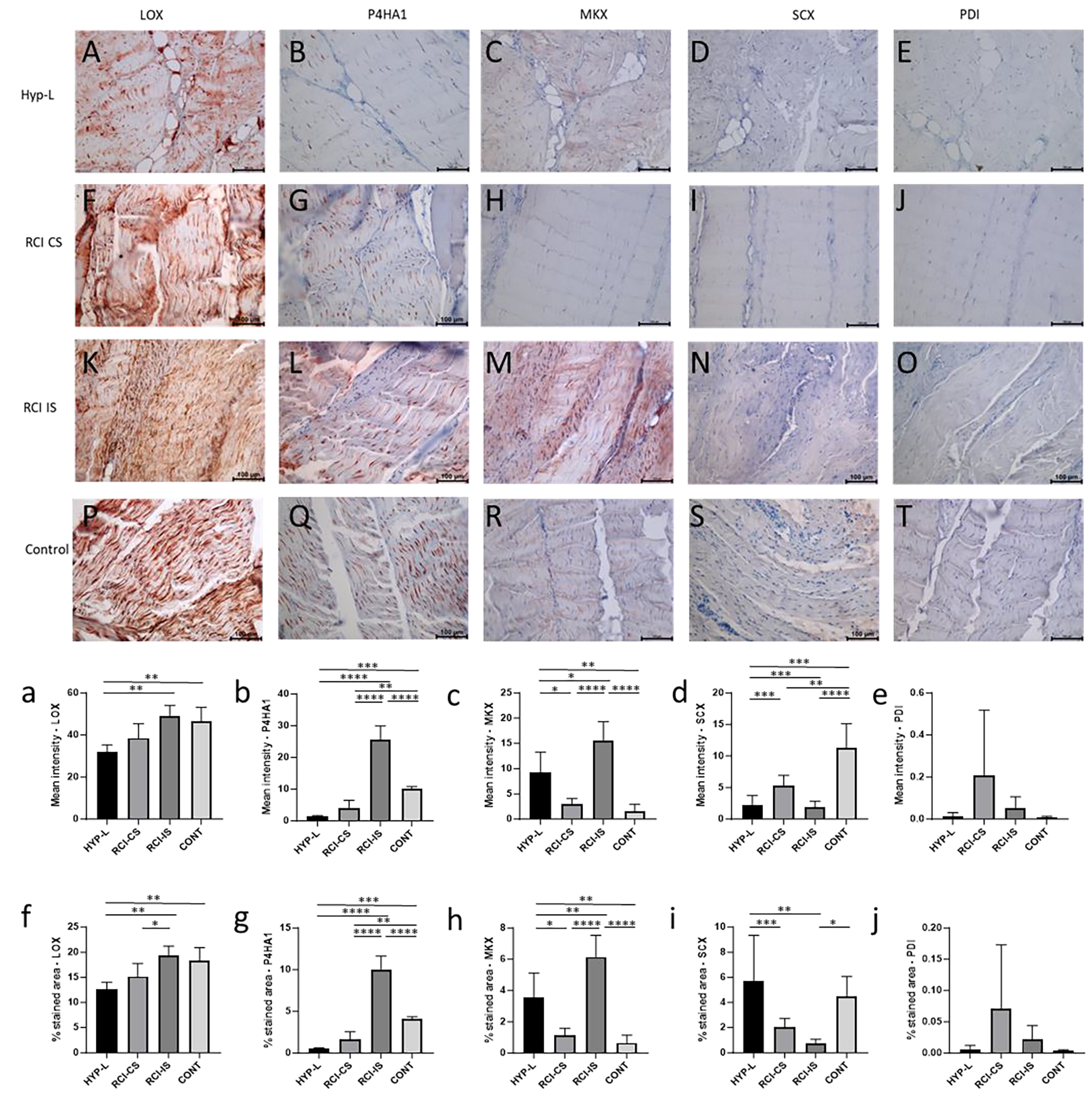
Representative image of immunostaining of infraspinatus tendon for LOX (A,F,K,P), P4HA1 (B,G,L,Q), MKX (C,H,M,R), SCX (D,I,N,S) and PDI (E,J,O,T). Mean intensity of staining and % area of staining of LOX (a,f), P2HA1 (b,g), MKX (c,h), SCX (d,i) and PDI (e,j). HYP-L indicates group of swines that received cholesterol-high-fat diet but no surgery. RCI-CS indicates uninjured tendon from contralateral side of swine that underwent either injury or injury + repair surgery and cholesterol-high-fat diet. RCI-IS indicates tendon tissues that had either of the surgery. CONT indicates control group of swines that received normal diet and no surgery. The images were acquired using 20× objective. Values are shown as mean ± SD; n=3–4). * *P*<0.05, ** *P*<0.01, *** *P*<0.001, **** *P*<0.0001.

Immunostaining of infraspinatus tendon for lysyl oxidase (LOX), prolyl 4-hydroxylase (P4HA1), mohawk (MKX), scleraxis (SCX), and protein disulphide-isomerase (PDI) are shown in Figure 2. All the group showed positive staining to LOX (Figure 2A, 2F, 2K and 2P). The mean intensity of staining for LOX was high in RCI-IS group of tissues while HYP-L group of tissues has low positivity staining. The difference in mean staining and % area of staining between the HYP-L group of tissues and control were significant (Figure 1a and 1f). P4HA1 showed differential expression among different group of infraspinatus tendon tissues (Figure 2B, 2G, 2L and 2Q). Mean intensity of staining for P4HA1 was higher in the RCI-IS tendon tissues while the control tendon tissues had the lowest staining. The positive staining for P4HA1 in the RCI-IS group was significantly higher than RCI-CS and control groups (Figure 2b and 2g) but was lower in the HYP-L group of tissues.

Positive staining for mohawk varied among groups with injury tendons (RCI-IS) showing maximum staining while the HYP-L showed lowest positive staining (Figure 2c and 2h). Positivity of MKX in control infraspinatus tendon tissues were higher than the HYP-L and RCI-CS tissues but were lower than the injured tendons (RCI-CS). Meanwhile positive staining for scleraxis was observed to be higher in the control group of tissues than other tissues. The mean intensity of staining was different between HYP-L, RCI-CS, and RCI-IS, but lower than tha control tendon tissues (Figure 2d and 2i). Meanwhile the positivity of protein disulphide-isomerase was very less or negligible in all the group of swines as observed from the mean intensity and % area stained (Figure 2e and 2j).

### 3.2 Immunostaining of Achilles tendon

**Figure 3:**
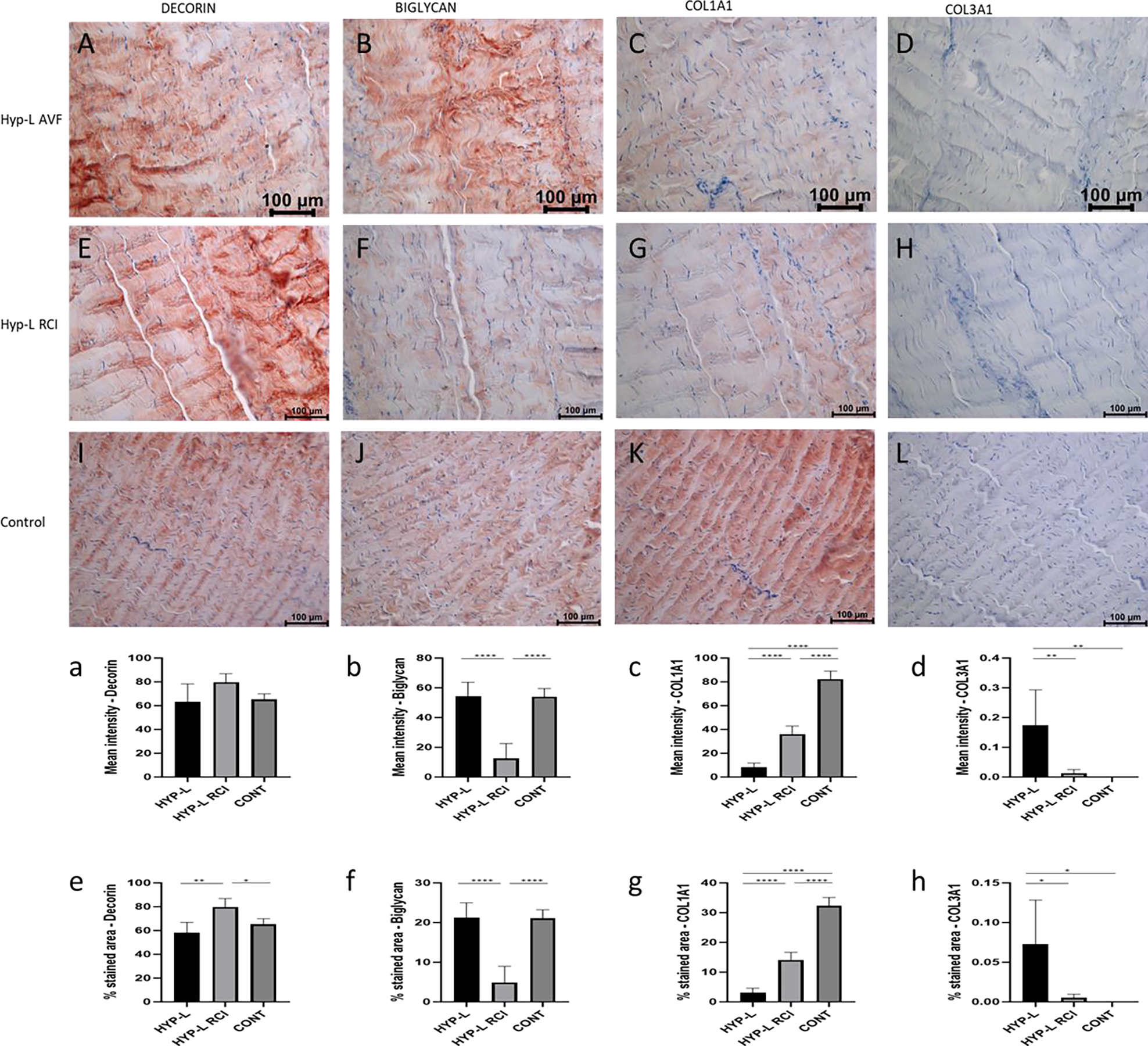
Representative image of immunostaining of Achilles tendon for decorin (A,E,I), biglycan (B,F,J), COL1A1 (C,G,K), and COL3A1 (D,H,L). Mean intensity of staining and % area of staining of decorin (a,e), biglycan (b,f), COL1A1 (c,g), and COL3A1 (d,h). HYP-L indicates group of swines that received cholesterol-high-fat diet but no surgery. HYP-L RCI indicates uninjured Achilles tendon of the swines underwent either injury or injury + repair surgery to the infraspinatus tendon and cholesterol-high-fat diet. CONT indicates control group of swines that received normal diet and no surgery. The images were acquired using 20x objective. Values are shown as mean ± SD; n=3–4). * *P*<0.05, ** *P*<0.01, *** *P*<0.001, **** *P*<0.0001.

Immunostaining of Achilles tendon for decorin, biglycan, COL1A1, and COL3A1 are shown in Figure 3. Positivity to decorin was observed in all the Achilles tendons tissues (Figure 3A, 3E and 3I). The mean intensity of staining for decorin in the Achilles tendon was not different between the control, HYP-L and swines that undergone injury or injury + repair surgeries in their infraspinatus tendon of the rotator cuff (HYP-L RCI). But the %area of staining was higher in the HYP-L RCI group of swine (Figure 3a and 3e). Positive staining for biglycan was observed in all three groups of tissues (Figure 3B, 3F and 3J). The mean intensity of staining and % area stained for biglycan in the HYP-l and control group of tissues were similar, but the above values for HYP-L RCI were significantly lower than other two groups (Figure 3b and 3f).

Positivity for COL1A1 staining was different between HYP-L, HYP-L RCI and the control group of Achilles tendon tissues (Figure 3C, 3G and 3K). Mean intensity of staining and % area of staining for COL1A1 were higher in the control tissues and lower in the HYP-L RCI and HYP-L Achilles tendon tissues (Figure 3c and 3g). HYP-L group of achilles tissues had the highest COL3A1 positive staining than the HYP-L RCI and control groups. Even though the mean intensity and % area of staining are significantly different the values were very low.

**Figure 4:**
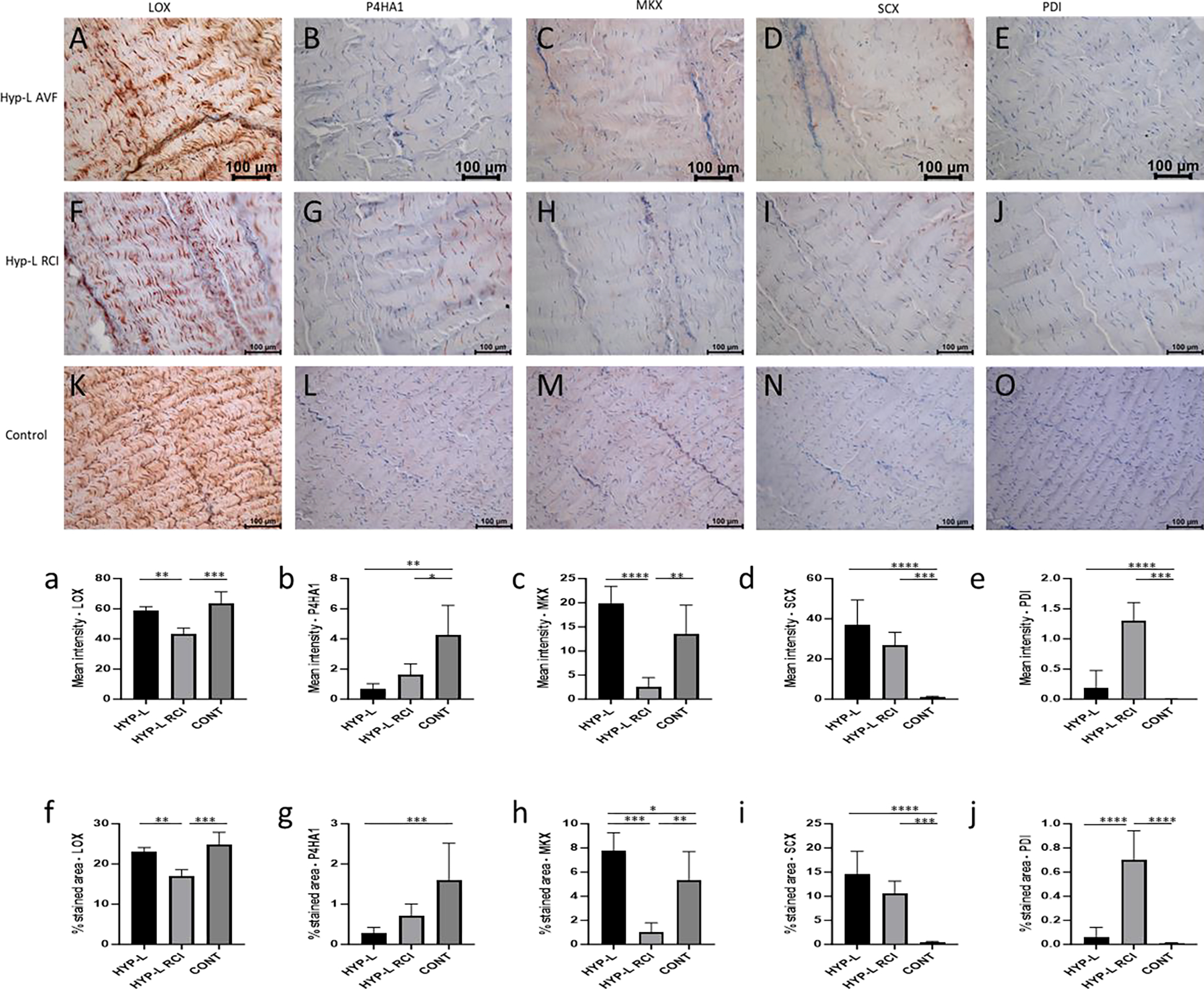
Representative image of immunostaining of Achilles tendon for LOX (A,F,K), P4HA1 (B,G,L), MKX (C,H,M), SCX (D,I,N) and PDI (E,J,O). Mean intensity of staining and % area of staining of LOX (a,f), P2HA1 (b,g), MKX (c,h), SCX (d,i) and PDI (e,j). HYP-L indicates group of swines that received cholesterol-high-fat diet but no surgery. HYP-L RCI indicates uninjured Achilles tendon of the swines underwent either injury or injury + repair surgery to the infraspinatus tendon and cholesterol-high-fat diet. CONT indicates control group of swines that received normal diet and no surgery. The images were acquired using 20× objective. Values are shown as mean ± SD; n=3–4). * *P*<0.05, ** *P*<0.01, *** *P*<0.001, **** *P*<0.0001.

Immunostaining of Achilles tendon for LOX, P4HA1, MKX, SCX and PDI are shown in Figure 4. Positivity for LOX was observed in all the three groups of swine tissues (Figure 4A, 4F and 4K). Mean intensity of staining and % area of staining of the HYP-L RCI group of tendon tissues were comparatively lower than the HYP-L and control tissues. Meanwhile there was no difference in mean intensity and % area of staining between the HYP-L and the control group of tissues (Figure 4a and 4f). Similar observation was found in positive staining for P4HA1 (Figure 4B, 4G and 4L). The semi quantitative analysis of mean intensity of staining revealed that the HYP-L RCI group of Achilles tendon had very low positive staining for P4HA1, while there was no significant difference in mean intensity of staining between HYP-L and control group (Figure 4b). On the other hand, % area staining was significantly different between the three groups (Figure 4g).

Positive staining for MKX was observed on all three groups (Figure 4C, 4H and 4M). The mean intensity of staining of the HYP-L group of tissues was lowest while the control tissues had the highest mean intensity of staining. Mean intensity of staining for MKX in the HYP-L RCI group of tissues was higher than the HYP-L group of tissues but less than the control (Figure 4c and 4h). On the other hand, the positive staining for SCX was very low and negligible in the control group of tissues when compared to that of HYP-L RCI and HYP-L group of tissues. HYP-L group of tissues had the higher mean intensity of staining and % area of staining (Figure 4d and 4i). Positive staining of PDI was significantly different between groups but the mean intensity and % area of staining had very low values (Figure 4e and 4j).

## 4. Discussion

Hyperlipidemia is a risk factor in tear and retear of rotator cuff tendons in elderly patients. Overall, our data indicates the role of tendon ECM component that contribute to the biomechanical properties of the tendons. The composition of the tendon ECM is important in maintenance of its structure and biomechanical properties [1]. We studied the expression of ECM proteoglycan (decorin, biglycan), structural proteins (COL1A1, COL3A1), enzymes in the formation and maintenance of tendon ECM (LOX, P4HA1 and PDI), and transcription factors that are activated by dynamic stretching and loading of the tendons (MKX and SCX) and aid in maintenance tendon tissue [2,14–17].

Decorin and biglycan is small leucin rich repeat proteoglycan that binds to the collagen fibrils and are attached to the glycosaminoglycans [18]. Decorin is involved in stabilization of inter-fibrillar organization of collagen fibrils [19]. Biglycan plays an important role in the collagen fiber diameter of collagen. In our study decorin was more expressed in infraspinatus tendon tissues of control tissues than the tendons that were injured (RCI-IS, P<0.001) and hyperlipidemia tissues that did not have any surgery (HYP-L, P<0.05). Conversely the biglycan was more in the infraspinatus tendon of the RCI-IS group than its contralateral tendon or control group (P<0.01). Meanwhile COL1A1 was also increased in the RCI-IS infraspinatus tendon. Our results contradicted with the previous results from Leiphart et al. [20], and Beach et al. [21], in mice models. They have reported that in patella tendon deficiency of biglycan led to the reduction of collagen fiber diameter [20] which reduced the dynamic modulus, stress relaxation while increasing the fiber realignment during loading [21]. The Achilles tendon of the swines that underwent surgical injury had experienced higher load and on the Achilles tendon but had very low biglycan staining compared to the control swines (P<0.0001) and Hyperlipidemic swines that did not had any surgeries (P<0.001). Previously we have reported that the hyperlipidemia swines which had injury on the infraspinatus tendon had very low dynamic modulus, ultimate tensile strength (UTS) and an increased strain % at break [10]. Hence COL1A1, decorin and biglycan are not the lone factors which decide the fate of tendon maintenance turnover. Another factor to consider is that the composition tendon is highly variable between tendons (say-Achilles, patella, etc.) and species and is directly related to the amount of load the tendon tissue is subjected to [22]. Achilles tendon of the control group had the most COL1A1 staining than the HYP-L group (P<0.0001) and HYP-L RCI group (P<0.0001) of swines. A previous studies conducted on 216 humans who received Achilles tendon rupture (ATR) surgery had higher cholesterol, triglyceride, and LDL levels than healthy people [23]. In our study the Achilles tendon on of the injury group of swines (HYP-L RCI) had more COL1A1 than the HYP-L group of swines. This could be the chronic adaptation to excessive loading on the Achilles tendon due to the injury in the rotator cuff [24].

Lysyl oxidase and Prolyl 4-hydroxylase are key factors in new tissue formation and in turn by crosslinking the collagen molecules and thus aid in mechanical properties [25]. The increase in LOX and P4HA1 in the infraspinatus tendons of the injury group (RCI-IS) indicates the active remodeling of the tendon after injury. In a previous study the mechanical properties of chick calcaneal tendon showed improved mechanical properties upon treatment with 3μg of lysyl oxidase [26]. LOX was low in the Achilles tendon of the HYP-L-RCI group of tissues than the HYP-L (P<0.01) and the control (P<0.01) group of tissues.

MKX was expressed more in the injury group of infraspinatus tendon tissues than the control tissues (P<0.0001), while lower in the Achilles tenson of the same groups (P<0.05). On the other hand, the SCX was more in the control group than the RCI-IS group (P<0.0001). MKX and SCX are transcription factors that are responsible for stretch activated remodeling and maintenance of tendons by sensing the load on the tissues [27]. The Achilles tendon subjected to chronic excess loading due to the injury on the infraspinatus tendon (HYP-L RCI group) would have activated the stretch cascade pathway and hence better biomechanical properties related factors were expressed [28–30]. The chronic adaptation of Achilles tendon to excess load due to the injury in the infraspinatus tendon would have been possible by the activation of stretch activated cascade of signaling which in turn would have contributed to the ECM of the tendon tissues.

## 5. Conclusion

This study highlights the changes in key components of tendon ECM and other factors involved in the maintenance and remodeling of infraspinatus tendon subjected to surgical injury and the chronic adaptation of the Achilles tendon to the excess load. Further detailed studies on biomechanical properties are warranted to understand the mechanism by which the tendon maintenance and turnover of ECM varies in hyperlipidemia and injury in large animals to identify new targets for treatment.
